# Performance Comparison of Systemic Inflammatory Response Syndrome with Logistic Regression Models to Predict Sepsis in Neonates

**DOI:** 10.3390/children4120111

**Published:** 2017-12-19

**Authors:** Jyoti Thakur, Sharvan Kumar Pahuja, Roop Pahuja

**Affiliations:** Department of Instrumentation and Control Engineering, Dr. B. R. Ambedkar National Institute of Technology, Jalandhar, Punjab 144011, India; pahujas@nitj.ac.in (S.K.P.); pahujar@nitj.ac.in (R.P.)

**Keywords:** neonates, sepsis, MIMIC III, SIRS, mobile application, international pediatric sepsis consensus conference

## Abstract

In 2005, an international pediatric sepsis consensus conference defined systemic inflammatory response syndrome (SIRS) for children <18 years of age, but excluded premature infants. In 2012, Hofer et al. investigated the predictive power of SIRS for term neonates. In this paper, we examined the accuracy of SIRS in predicting sepsis in neonates, irrespective of their gestational age (i.e., pre-term, term, and post-term). We also created two prediction models, named Model A and Model B, using binary logistic regression. Both models performed better than SIRS. We also developed an android application so that physicians can easily use Model A and Model B in real-world scenarios. The sensitivity, specificity, positive likelihood ratio (PLR) and negative likelihood ratio (NLR) in cases of SIRS were 16.15%, 95.53%, 3.61, and 0.88, respectively, whereas they were 29.17%, 97.82%, 13.36, and 0.72, respectively, in the case of Model A, and 31.25%, 97.30%, 11.56, and 0.71, respectively, in the case of Model B. All models were significant with *p* < 0.001.

## 1. Introduction

Sepsis is the major cause of deaths in neonates, and most of these deaths occur in countries that lack resources [1,2]. Due to non-specific clinical signs, diagnosis of bacterial sepsis remains a difficult task for neonatologists [3]. Blood culture remains the gold standard for identifying sepsis. Although, its accuracy is not 100%, a positive blood culture may be due to presence of contaminants in blood [4]. In addition, a negative blood culture result may be due to a low volume of blood [5,6]. Despite this, whenever there is a suspicion of sepsis, blood is drawn for blood culture, and the neonate is started on antibiotics. For each neonate with blood culture-proven sepsis, approximately 12 additional newborns receive antibiotics, resulting in bacterial resistance and excessive hospital costs [7,8,9,10].

The definition of sepsis has been revised three times—in 1992 [11], 2001 [12], and in 2016 [13]—but none of these definitions defines sepsis in the case of neonates. In 2005, a consensus definition of sepsis was determined for all children less than 18 years of age, and pediatric systemic inflammatory response syndrome (SIRS) was defined; however, preterm neonates (<37 weeks of gestation) were excluded from this definition [14]. In 2012, Nora Hofer et al. [15] did a study to check the performance of the SIRS definition given in 2005, but did not include preterm neonates. 

In this paper, we tested the capability of SIRS to predict sepsis in neonates irrespective of their gestational age (i.e., pre-term, term, or post-term), using the cut-off values given in [14]. We also made two prediction models, named Model A and Model B, using logistic regression. Model A consisted of the same parameters as those of SIRS. Model B included birth weight as an additional independent variable (in addition to the Model A parameters). Our decision to add birth weight was based on a conclusion derived from statistical analysis (refer to the Results section), in addition to statements given in [16] (“The risk of late-onset sepsis increases with decreasing birth weight and gestational age.”) and [17] (“Early onset sepsis is an important cause of illness and death among infants with very low birth weights (less than 1500 g)”). To use these models in real-world scenarios, we made an android application that does all of the complex calculations, and can be easily used by physicians.

## 2. Materials and Methods

### 2.1. Study Design

This is a retrospective study, which uses the data from the Medical Information Mart for Intensive care (MIMIC) III dataset [18,19]. MIMIC III is an open-access research database that contains more than 58,000 hospital admissions for 38,645 adults and 7875 neonates. The data was collected from the intensive care units at Beth Israel Deaconess Medical center (BIDMC) between June 2001 and October 2012. All data were de-identified in accordance with Health Insurance Portability and Accountability Act (HIPAA); therefore, patient consent was waived by the Institutional review boards of Massachusetts Institute of Technology (MIT) and BIDMC.

Inclusion criteria were the presence of blood culture report and age ≤30 days at the time of the first phlebotomy for blood culture. Neonates with missing or incomplete data necessary for the calculation of scores were excluded from the study.

### 2.2. Definitions

We defined sepsis as the positive blood and/or Cerebrospinal Fluid (CSF) culture [20,21]. The time of suspicion of sepsis was defined as the initial time of the earliest culture draw. We defined a time window of 12 h starting from the suspicion of sepsis, and all the required parameters were taken in this time window.

### 2.3. SIRS

Table 1 shows the cut-off values of SIRS parameters mentioned in the pediatric consensus definition of SIRS [14]. It can be seen that cut-off values vary with age. According to this definition, at least two of the following four criteria must be met, and one of which must be abnormal temperature or abnormal leukocyte count, although some literature shows that white blood cell (WBC) count and temperature shows less sensitivity in the diagnosis of neonatal sepsis [22,23].

### 2.4. Statistical Analysis

All the data was extracted from the MIMIC III dataset using Postgre SQL 9.6 queries (Global Development Group, Berkeley, CA, USA). We used IBM SPSS Statistics version 24 (SPSS Inc., Chicago, IL, USA) and Microsoft Excel 2016 (Microsoft, Inc., Redmond, WA, USA) for the statistical analysis. Sensitivity, specificity, positive predictive value (PPV), negative predictive value (NPV), positive likelihood ratio (PLR), and negative likelihood ratio (NLR) were calculated for each model. Fisher’s Exact Test and Pearson’s Chi-Square Test were used as a goodness-of-fit tests. We used *p* < 0.001 to determine statistical significance.

**Figure 1:**
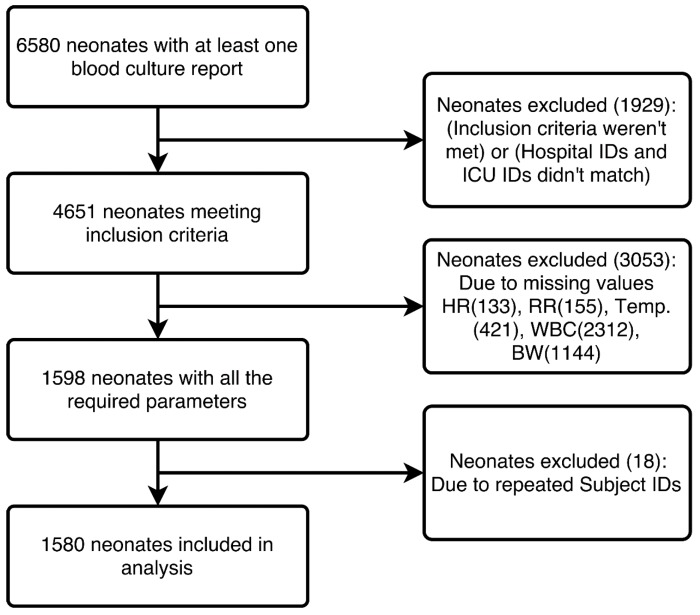
Study dataset generation from the Medical Information Mart for Intensive Care III (MIMIC III) database.

Among the 4651 neonates that met the inclusion criteria, 3053 neonates were excluded because of incomplete data, and 18 were excluded because of duplicate entries. Thus, 1580 neonates with 204 cases of sepsis (12.91% disease prevalence) were studied. Figure 1 shows the complete extraction process from the MIMIC III database.

We extracted a total of 9 parameters within the time window of 12 h (as mentioned above), including blood culture reports, CSF culture, temperature (Temp.), heart rate (HR), respiration rate (RR), WBC count, birth weight, age at the time of admission, and gestational age. All the parameters were extracted using PostgreSQL queries except gestational age, which was extracted manually from text notes available in the MIMIC III dataset. Minimum and maximum values of these parameters (wherever applicable) were calculated. Table 2 shows the baseline characteristics of 1580 neonates.

## 3. Results

To select the independent variables for Model A, we used binary logistic regression using backward logistic regression (LR) and discriminant analysis, using the stepwise method in SPSS. Both methods resulted in the exclusion of Respiratory Rate (Maximum), as well as Temperature (Maximum). The remaining parameters, as shown in Table 3 (excluding birth weight), were used to build Model A. Table 3 also shows the predictive power of independent variables in decreasing order, using a structure matrix.

For Model B, we used the Omnibus tests of the model coefficients to examine the significance of birth weight as an additional parameter. Table 4 shows the Chi-Square values with *p* < 0.001 in Model B, with Model A used as a baseline model and birth weight being added as an additional parameter. This table shows that Model B provides a better fit for the prediction of neonatal sepsis.

Thus, two models were created using binary logistic regression. Model A consisted of five variables (heart rate, respiratory rate, temperature, leukocyte count, and age at the time of first culture draw) that were then also used to validate SIRS. The main difference between SIRS and Model A is that SIRS is calculated using a simple decision rule that uses the cut-off values mentioned in Table 1. Whereas Model A was developed using logistic regression; it predicts the outcome as a probability, rather than as a binary decision. To predict the probability of sepsis, predictor variables are entered, and the probability is calculated using the steps shown in Section: Mobile Application of this paper.

In Model B, we included birth weight as a fifth parameter. For Model A and Model B, the data were randomly divided into two parts, i.e., 70/30, for training and validation purposes. These two models were trained and validated, and were compared with the SIRS. Table 5 shows the comparison of these two models with SIRS. Table 5 illustrates that Model A performed better than SIRS, despite using the same parameters. Moreover, Model B showed better sensitivity than Model A. Both of these models were compared at a 0.5 cut-off value.

Table 6 shows the goodness-of-fit test results for the three prediction models using the Pearson Chi-Square and Fisher’s Exact Test (whichever is applicable). All the models were statistically significant with *p* < 0.001.

Table 7 shows the performance of Model A and Model B at different cut-off values of probability. This table helps the reader to gain a better understanding of what to expect from each of these two models with respect to sensitivity and specificity. As expected, the value for sensitivity decreases as we move from a lower cut-off value to a higher cut off value. At a cut-off value of 0.7, the sensitivity and specificity of Model A and Model B are approximately equal to SIRS. Hence, the usefulness of both Model A and Model B vanishes.

### Mobile Application

To use Model A and Model B in place of SIRS, it is necessary to make its implementation simpler in the real world. To this end, we created an android application that can predict sepsis in neonates using all three models; namely, SIRS, Model A and Model B.

**Figure 2:**
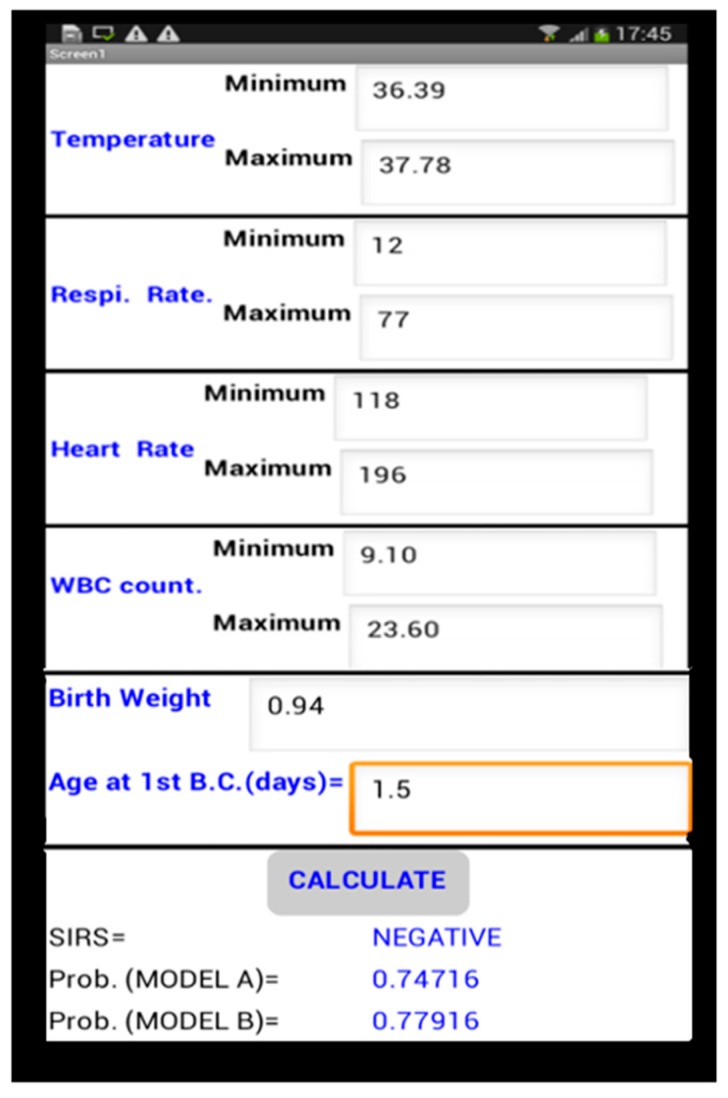
Screenshot of mobile application.

Figure 2 shows screenshots of the mobile application that we developed using the App Inventor platform [24]. App Inventor is an open-source visual programming environment that is maintained by MIT. In the bottom of Figure 2, we can see that there is a contradiction between the outcomes of the three models. As per the SIRS criteria, the neonate is free from sepsis, whereas the other two models show a high probability of sepsis. In this particular scenario, Model A and Model B correctly predicted the outcome (the data shown is of a sepsis-positive neonate).

**Figure 3:**
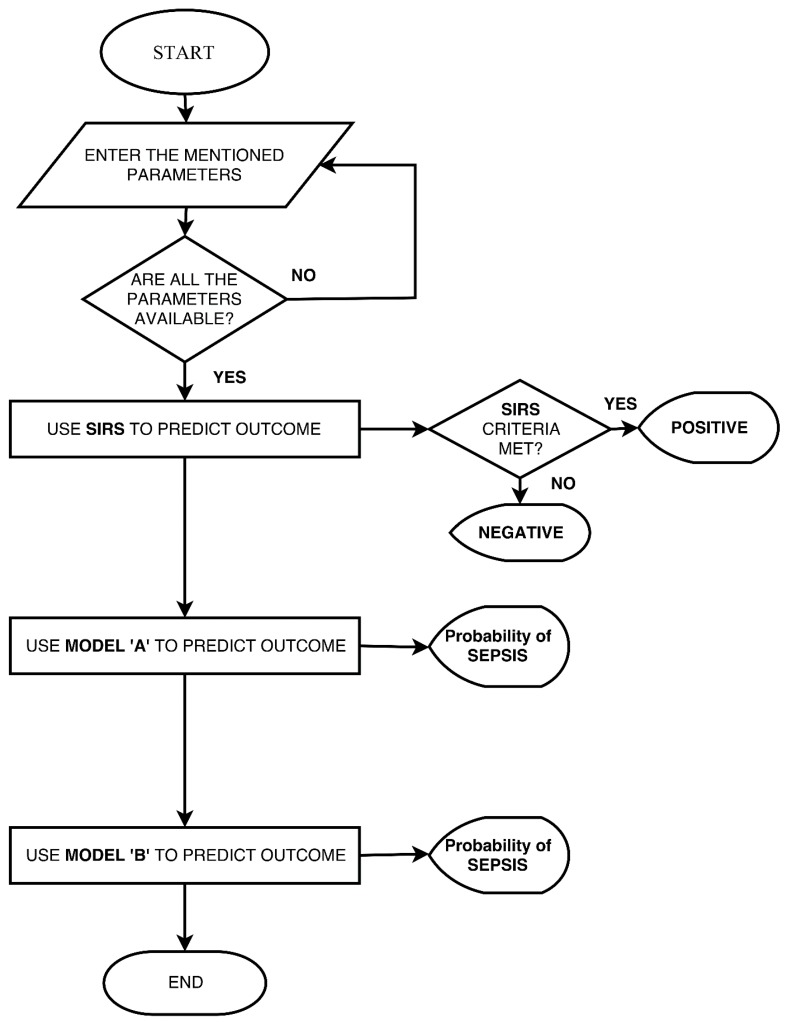
Flow chart showing the operation of the mobile application.

Figure 3 shows the algorithm for the mobile application. SIRS was calculated according to the cut-off values given in Table 1; while for Model A and Model B, the logistic regression coefficients were calculated using IBM SPSS, and were stored in the Android application. The steps shown below were used for the prediction of outcomes in Model A and Model B.

**Step 1**: $\mathrm{Calculate}\;\mathrm{Logit}\;\left(L\right)=\beta_{0}+\beta_{1}X_{1}+\beta_{2}X_{2}+\cdots +\beta_{3}X_{3}\;\left(i\right)$
  where *β*_0_, *β*_1_, ...., *β_n_* are the logistic regression coefficients and *X_1_*, *X_2_*, ..., *X_n_* are the independent predictor variables.
**Step 2**: $\text{Calculate}\;\text{Probability}\;\left(P\right)=\left(e^{L}\right)/\left(1+e^{L}\right)$
  where *L* is the Logit calculated in step 1.

The probability calculated in step 2 is displayed as the output of Model A and Model B.

## 4. Discussion

**Figure 4:**
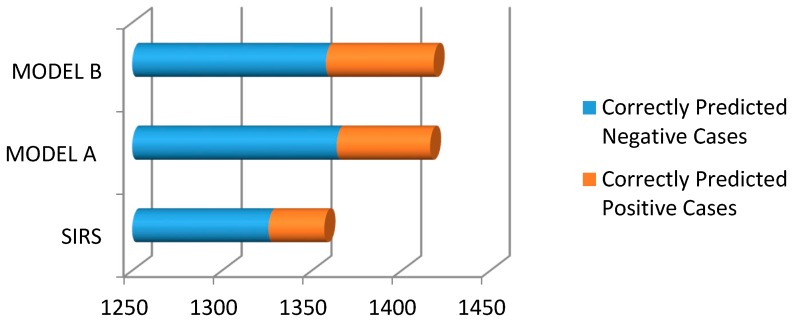
Bar chart showing the correct predictions made by the three models.

To date, the performance of the SIRS definition given by the pediatric sepsis consensus conference has not been tested in preterm neonates [25]. In this study, we tested SIRS accuracy in neonates irrespective of their gestational age, and compared it with the two prediction models (developed by us) using binary logistic regression. Both LR models performed better than SIRS. Figure 4 shows the performance of all three models in terms of correctly predicted positive and negative cases. For comparison, the cut-off values of Model A and Model B were taken as 0.5.

The calculations required to predict the probability of Model A and Model B are time-consuming and complex. We overcame this limitation by developing an android application; it does all the calculations and makes it easier for physicians to use complex prediction models.

Our study has several limitations. The values of the prediction variables have to be identified (maximum and minimum of the parameters) and entered manually in the mobile application. In future work, we will try to develop a system that can automatically enter non-invasive parameters values. Further improvements of models may be possible through the use of sophisticated machine-learning techniques, such as artificial neural networks (ANN).

These models can assist physicians in making the decision to start antibiotics in cases of sepsis-positive neonates and to stop antibiotics in sepsis-negative neonates before the blood culture report is available. This study also provides the hope of improving the performance of existing Clinical Decision Rules (CDRs) or scoring systems.

## 5. Conclusions

To confirm the results, both models should be evaluated prospectively and externally (using datasets from other sources) for predicting sepsis in neonates within 12 h of the first draw of blood culture. The sensitivity of SIRS in predicting sepsis in neonates (irrespective of their gestational age) was quite low. LR models outperformed SIRS in predicting neonatal sepsis, despite using the same parameters. Further improvements of these models could help the decision-making processes of physicians. We speculate that the same approach may improve the performance of other clinical decision rules and scoring systems.

## Figures and Tables

**Table 1:** Systemic inflammatory response syndrome (SIRS) cut-off values for neonates according to the International Pediatric Sepsis Consensus conference.

Neonatal Variables	Cut-off Values According to Age	
	Age 0 Days to 1 Week	Age 1 Week to 1 Month
Heart Rate (Beats/min)	>180 or <100	>180 or <100
Respiratory Rate (Breaths/min)	>50	>40
Leukocyte Count (×10^3^/mm^3^)	>34	>19.5 or <5
Temperature (°C)	>38.5 or <36	>38.5 or <36

**Table 2:** Baseline characteristics of neonates (*N* = 1580).

**Sex (Male/Female)**	**(915/665)**	
	**Mean**	**Range**
Birth Weight (kg)	2.18	(0.36–5.4)
Gestational Age (weeks)	32.99	(18–43)
**Features**	**Present In**	
	**N**	**%**
Blood Culture (BC)	(204/1376) ^†^	(12.91/87.08) ^†^
Cerebrospinal fluid (CSF) culture	(10/1570) ^†^	(0.63/99.36) ^†^
BC and/or CSF culture	(204/1376) ^†^	(12.91/87.08) ^†^
Mortality	28	1.77
Abnormal Heart Rate *	291	18.41
Abnormal Respiratory Rate *	1456	92.15
Abnormal Leukocyte count *	26	1.64
Abnormal Temperature *	74	4.68

^†^ Represents Positive/Negative; * As per International pediatrics sepsis consensus conference definition of SIRS 2005.

**Table 3:** Structure matrix showing the predictive power of independent variables.

Sr. No.	Name of the Parameter	Function 1
1	Birth Weight	0.633
2	Heart Rate (Maximum)	−0.625
3	Respiratory Rate (Minimum)	0.495
4	Temperature (Minimum)	0.403
5	WBC (Maximum)	−0.285
6	WBC (Minimum)	0.256
7	Age at first Blood culture draw	−0.135
8	Heart Rate (Minimum)	−0.100

WBC: white blood cell.

**Table 4:** Omnibus tests of model coefficients.

		Chi-Square	*d*f	Sig.
Step 1	Step	26.961	1	0.000
	Block	26.961	1	0.000
	Model	252.869	8	0.000

**Table 5:** Performance parameters of SIRS, Model A and Model B.

	Sensitivity	Specificity	PPV	NPV	PLR	NLR
SIRS	16.15	95.53	33.33	89.17	3.61	0.88
95% CI	(11.24–22.13)	(94.31–96.56)	(25.03–42.82)	(88.55–89.77)	(2.41–5.41)	(0.82–0.93)
Model A ^†^	29.17	97.82	66.67	90.22	13.36	0.72
95% CI	(21.90–37.32)	(96.68–98.64)	(54.97–76.62)	(89.25–91.11)	(8.16–21.89)	(0.65–0.80)
Model A *	20.83	99.30	76.92	91.76	29.58	0.80
95% CI	(10.47–34.99)	(97.96–99.85)	(48.72–92.12)	(90.59–92.79)	(8.43–103.80)	(0.69–0.92)
Model B ^†^	31.25	97.30	63.38	90.43	11.56	0.71
95% CI	(23.79–39.50)	(96.06–98.23)	(52.46–73.08)	(89.43–91.35)	(7.37–18.13)	(0.63–0.79)
Model B *	31.25	99.06	78.95	92.75	33.28	0.69
95% CI	(18.66–46.25)	(97.61–99.74)	(56.46–91.56)	(91.35–93.93)	(11.51–96.24)	(0.57–0.84)

^†^ Training Set; * Testing Set; PPV: Positive Predictive Value; NPV: Negative Predictive Value; PLR: Positive Likelihood ratio; NLR: Negative Likelihood ratio; CI: Confidence Interval.

**Table 6:** Goodness-of-fit test results using Pearson Chi-Square Test and Fisher’s Exact Test.

	Value	*d*f	Significance
SIRS ^‡^	41.530	1	<0.001
Model A ^†,‡^	169.774	1	<0.001
Model B ^†,‡^	169.912	1	<0.001
Model A *^,¥^	-	-	<0.001
Model B *^,¥^	-	-	<0.001

^†^ Training Set; * Testing Set; ^‡^ Pearson Chi-Square; ^¥^ Fisher’s Exact Test.

**Table 7:** Sensitivity and specificity of Model A and Model B at different cut-off values.

	Cut-Off	Prediction Performance (%)			
		Training Set		Testing Set	
		Sn	Sp	Sn	Sp
**Model A**	0.1	81.94 (74.67–87.85)	72.45 (69.51–75.26)	75.00 (60.40–86.36)	78.17 (73.94–82.00)
	0.2	67.36 (59.06–74.93)	89.60 (87.50–91.46)	68.75 (53.75–81.34)	92.02 (89.03–94.41)
	0.3	44.44 (36.17–52.95)	93.66 (91.93–95.12)	33.33 (20.40–48.41)	95.07 (92.56–96.92)
	0.4	34.72 (26.99–43.10)	95.95 (94.50–97.10)	25.00 (13.64–39.60)	98.83 (97.28–99.62)
	0.5	29.17 (21.90–37.32)	97.82 (96.68–98.64)	20.83 (10.47–34.99)	99.30 (97.96–99.85)
	0.6	22.22 (15.72–29.90)	98.75 (97.83–99.35)	16.67 (7.48–30.22)	99.30 (97.96–99.85)
	0.7	16.67 (10.98–23.78)	98.96 (98.10–99.50)	4.17 (0.51–14.25)	99.53 (98.31–99.94)
**Model B**	0.1	82.64 (75.45–88.44)	74.95 (72.08–77.66)	77.08 (62.69–87.97)	79.58 (75.43–83.31)
	0.2	68.75 (60.50–76.21)	88.67 (86.50–90.60)	66.67 (51.59–79.60)	91.08 (87.96–93.61)
	0.3	56.25 (47.74–64.49)	93.45 (91.70–94.93)	50.00 (35.23–64.77)	94.37 (91.73–96.36)
	0 4	41.67 (33.52–50.17)	95.43 (93.91–96.66)	41.67 (27.61–56.79)	97.65 (95.73–98.87)
	0.5	31.25 (23.79–39.50)	97.30 (96.06–98.23)	31.25 (18.66–46.25)	99.06 (97.61–99.74)
	0.6	24.31 (17.55–32.15)	98.44 (97.44–99.12)	18.75 (8.95–32.63)	99.30 (97.96–99.85)
	0.7	16.67 (10.98–23.78)	98.96 (98.10–99.50)	14.58 (6.07–27.76)	99.53 (98.31–99.94)

Values in parenthesis indicate 95% confidence interval. Sn: sensitivity; Sp: specificity.
